# Astroglial Knockout of Glucocorticoid Receptor Attenuates Morphine Withdrawal Symptoms, but Not Antinociception and Tolerance in Mice

**DOI:** 10.1007/s10571-021-01086-3

**Published:** 2021-04-05

**Authors:** Magdalena Tertil, Urszula Skupio, Lucja Kudla, Lucja Wiktorowska, Ryszard Przewlocki

**Affiliations:** 1 Laboratory of Pharmacology and Brain Biostructure, Department of Pharmacology, Maj Institute of Pharmacology Polish Academy of Sciences, Krakow, Poland; 2 Department of Molecular Neuropharmacology, Maj Institute of Pharmacology Polish Academy of Sciences, ul. Smetna 12, 31-343 Krakow, Poland

**Keywords:** Astrocytes, Glucocorticoid receptor, Morphine withdrawal, Morphine tolerance

## Abstract

The development of tolerance and drug dependence limit the clinical application of opioids for the treatment of severe pain. Glucocorticoid receptors (GRs) are among molecular substrates involved in these processes. Most studies focus on the role of neuronal GR, while the involvement of GR on glial cells is not fully understood. To address this issue, we used a transgenic model of conditional GR knockout mice, targeted to connexin 30-expressing astrocytes, treated with repeated doses of morphine. We observed no difference between control mice and astrocytic GR knockouts in the development of antinociceptive tolerance. Nevertheless, when animals were subjected to precipitated withdrawal, knockouts presented some attenuated symptoms, including jumping. Taken together, our data suggest that hippocampal and spinal astrocytic GRs appear to be involved in opioid withdrawal, and drugs targeting the GR may relieve some symptoms of morphine withdrawal without influencing its antinociceptive properties.

Opioids are frequently used drugs in the clinical management of severe pain. However, their application is limited by the development of analgesic tolerance and drug dependence following prolonged or repeated use. Accumulating evidence suggests that glucocorticoid receptors (GRs) are involved in the antinociceptive action of the drug and play a role in opioid-induced analgesia and tolerance [Wong et al. 1982, Lim et al. 2005a, Navarro-Zaragoza et al. 2012]. Most of the studies focus on mechanisms related to the neuronal GR, while the role of GR on glial cells is not fully understood, although there are clear indications of the involvement of GR-responsive genes, such as aquaporin 4 and serum and glucocorticoid-regulated kinase 1 in astrocytes in these processes [Wu et al. 2008, Xiao et al. 2019]. Our previous research demonstrated that astrocytes are a major cellular target of the transcriptional component of morphine action via GR [Slezak et al. 2013] and that astrocytic GR activation is involved in morphine reward [Skupio et al. 2020]. Yet it has not been investigated whether both glial cells and GRs may play a role in opioid-induced antinociception and tolerance/withdrawal.

To address these questions, we have employed a murine-targeted GR knockout model. The conditional astrocytic deletion of GR was achieved by crossing GR flox/flox animals with a transgenic strain expressing CreERT2 under connexin-30 promoter (GR^astroKO^), as previously described [Tertil et al. 2018]. The mRNA levels of GR in the lumbar spinal cord (SC) were reduced by approximately 30% within the whole tissue lysate, indicating a high inactivation of the GR allele in this area in comparison with other regions of the central nervous system (Fig. 1a). It was further corroborated by the pattern of induction of a GR-targeted gene *Fkbp5* after the injection of a GR agonist dexamethasone (Dex), which was reduced by over 60% in the spinal cord (Fig. 1b). The amygdala and hippocampus also showed an impaired GR transcriptional response, confirming the previously presented validation of the model [Tertil et al. 2018]. Importantly, when we measured the regulation of an astrocyte-specific GR target gene *Gjb6* (coding for Cx30) in the lumbar SC, no significant upregulation could be observed in GR^astroKO^ animals injected with Dex, indicating the efficient inactivation of the GR-dependent transcriptional response within this cell type (Fig. 1c).

**Fig.1:**
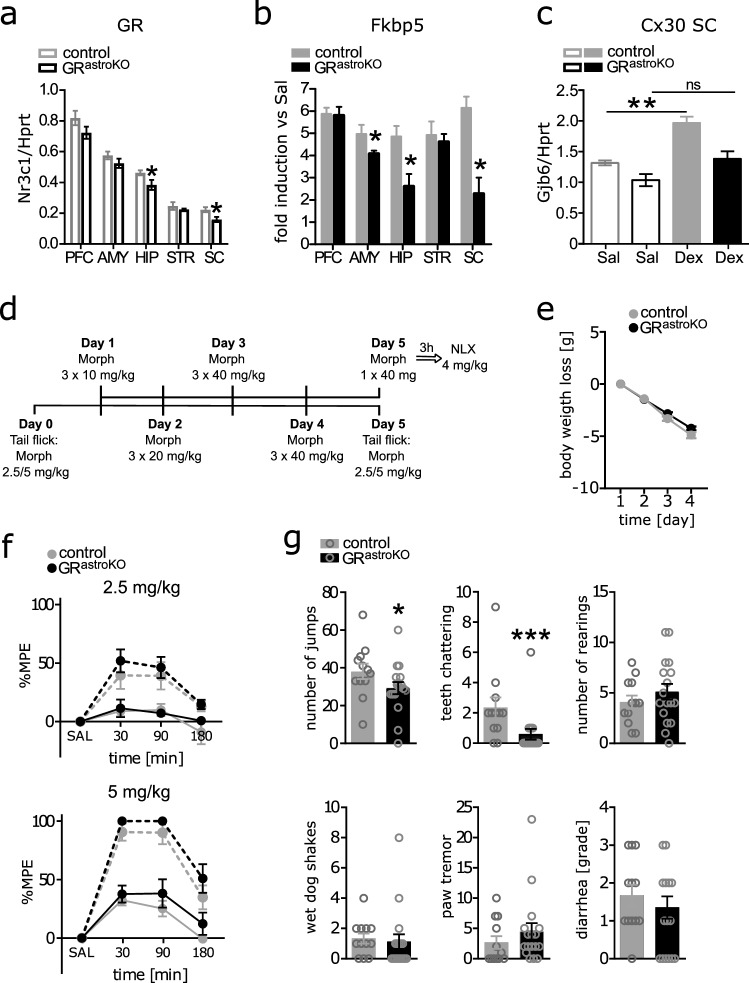
**a** Expression of *Nr3c1* (GR) mRNA normalized to reference gene *Hprt* determined by real-time qPCR in prefrontal cortex (PFC, t_6_ = 1.56, *p* = 0.09), amygdala (AMY, t_6_ = 1.32, *p* = 0.12), hippocampus (HIP, t_6_ = 2.17, *p* = 0.04), striatum (STR, t_6_ = 0.89, *p* = 0.21), and lumbar spinal cord (SC, t_6_ = 2.73, *p* = 0.02) of control (*n* = 4) and GR knockout (*n* = 4) animals. **b** Relative induction of GR target gene *Fkbp5* (PFC: t_7_ = 0.13 p = 0.45; AMY: t_7_ = 2.29 *p* = 0.03; HIP: t_7_ = 2.99 *p* = 0.01; STR: t_7_ = 0.43 *p* = 0.34; SC: t_7_ = 4.16 *p* = 0.002) in different regions of the central nervous system 4 h following i.p. injection of 4 mg/kg GR agonist dexamethasone: qPCR data normalized by ΔΔCt method to control animals receiving saline. **c** Regulation of transcription of Cx30 in lumbar SC following treatment with Dex (as in **b**) – levels of *Gjb6* mRNA normalized to reference gene *Hprt* determined by real-time qPCR (genotype effect: F_1,12_ = 17.42, *p* < 0.001; treatment effect: F_1,12_ = 20.72, *p* < 0.001). **d** Scheme of the administration of morphine for the investigation of naloxone-precipitated withdrawal and drug tolerance. **e** Body weight loss during each day of morphine administration in control and GR knockout mice (*n* = 19/genotype, time effect F_3,114_ = 451, *p* < 0.001, genotype effect insignificant). **f** Results of tail-flick test (maximum possible effect- %MPE) – antinociceptive effect of two doses of morphine (*n* = 10/genotype for 2.5 mg/kg, F_7,126_ = 0.32, *p* = 0.94, time effect: F_7,126_ = 16.74, *p* < 0.001, genotype effect insignificant; *n* = 9/genotype for 5 mg/kg, F_7,112_ = 0.46, *p* = 0.86, time effect: F_7,112_ = 75.71, *p* < 0.001, genotype effect insignificant) after acute (dotted lines) and chronic (solid lines) administration of the drug. **g** Evaluation of symptoms of precipitated withdrawal in control (*n* = 12) and GR knockout (*n* = 17) mice (jumps: U = 55, *p* = 0.03 teeth chattering: U = 35, *p* < 0.001; rearings: t_27_ = 0.96, *p* = 0.34; wet dog shakes: U = 71, *p* = 0.15; paw tremor: U = 76, *p* = 0.25; diarrhea: U = 85.5, *p* = 0.45). All data represent mean ± SEM. (**a**, **b**, **g**) Unpaired Student’s t-test or Mann–Whitney test (when data distribution did not pass Anderson–Darling normality test), (**c**) two-way ANOVA followed by Bonferroni post hoc analysis; (**e**, **f**) two-way repeated measures ANOVA

To model chronic morphine exposure, mice received 3 daily injections of the drug in increasing doses for 4 days (Fig. 1d). We noted similar weight loss in both control and GR^astroKO^ mice (Fig. 1e). To investigate the development of tolerance, pain responses after a 2.5 mg/kg or 5 mg/kg morphine injection were measured one day prior to and one day after the chronic morphine administration (Fig. 1d). Baseline tail-flick measurements were performed 15 min before the morphine injection and confirmed no differences between genotypes in basal pain sensitivity already demonstrated in our previous study [Tertil et al. 2018]. The animals were tested for antinociception 30, 90, and 180 min after a single i.p. morphine administration (Analgesia Meter; Ugo Basile). Both control and GR^astroKO^ mice presented similar analgesic responses to the two different doses of morphine tested (Fig. 1f). The chronic administration of the drug resulted in the development of tolerance to its antinociceptive action in both groups of animals regardless of the genotype. This is in contrast to previously published data, which showed that non-cell type-specific blocking of spinal GR by inhibition of its expression or co-infusion of the receptor antagonist attenuated this phenomenon [Lim et al. 2005b, Zhai et al. 2018]. Thus, our data suggest that GR-dependent transcriptional responses to morphine in Cx30-positive astroglia are not involved in the development of tolerance to the drug, pointing rather to a neuron-dependent mechanism.

Another crucial limitation in the clinical application of morphine-based analgesics is the development of physical dependence, and GR has been widely implicated as one of the molecular substrates of addictive properties of drugs of abuse. Therefore, we next tested naloxone-precipitated opioid withdrawal symptoms after chronic morphine treatment (scheme as in Fig. 1d) in our transgenic model using previously described protocol [Parkitna et al. 2012]. Saline-receiving control groups were not introduced as it has been already demonstrated that saline-treated mice do not develop withdrawal syndrome [Solecki et al. 2019]. On the last day, mice received a single injection of 40 mg/kg morphine followed by an injection of naloxone (4 mg/kg) after 3 h and withdrawal symptoms were evaluated immediately. Jumping and teeth chattering were significantly decreased in GR^astroKO^ mice (Fig. 1g). This attenuation of the somatic expression of withdrawal directly confirms a role of astrocytic GR in morphine addiction that could be hypothesized from previous studies utilizing the non-specific inhibition of glial cell metabolism or pharmacological antagonists of GR [Navarro-Zaragoza et al. 2012, Seyedaghamiri et al. 2018]. It has to be noted that other withdrawal symptoms remained similar in both control and GR^astroKO^ mice, which may perhaps be due to the limited efficiency of our Cx30-CreERT2-driven knockout model, as demonstrated by the gene expression data. Spinal cord-associated jumping was attenuated while rearing, wet dog shakes and paw tremor were not significantly altered. The observed effects could also be at least partly associated with GRs localized in Cx30-positive glia in the hippocampus, where the targeting of our model is evident [Tertil et al. 2018], in line with the observations of Seyedaghamiri (2018) showing effects of local glial inhibition in the CA1 region.

Given the postulated role of neuronal GR in opiate addiction, our data suggest that general, non-cell type-specific inhibition of GR may be the right strategy to ameliorate the aversive symptoms of opiate withdrawal.

## Data Availability

The datasets used and/or analyzed during the current study are available from the corresponding author on reasonable request.
